# Nicotine Levels and Presence of Selected Tobacco-Derived Toxins in Tobacco Flavoured Electronic Cigarette Refill Liquids

**DOI:** 10.3390/ijerph120403439

**Published:** 2015-03-24

**Authors:** Konstantinos E. Farsalinos, I. Gene Gillman, Matt S. Melvin, Amelia R. Paolantonio, Wendy J. Gardow, Kathy E. Humphries, Sherri E. Brown, Konstantinos Poulas, Vassilis Voudris

**Affiliations:** 1 Department of Cardiology, Onassis Cardiac Surgery Center, Kallithea 17674, Greece; E-Mail: vvoudris@otenet.gr; 2 Department of Pharmacy, University of Patras, Rio 26500, Greece; E-Mail: kpoulas@otenet.gr; 3 Enthalpy Analytical Inc., Durham NC 27713, USA; E-Mails: Gene.Gillman@enthalpy.com (I.G.G.); MMelvin@lortobco.com (M.S.M.); Amelia.Paolantonio@enthalpy.com (A.R.P.); Wendy.Gardow@enthalpy.com (W.J.G.); Kathy.Humphries@enthalpy.com (K.E.H.); Sherri.Brown@enthalpy.com (S.E.B.)

**Keywords:** electronic cigarette, tobacco, nitrosamines, aldehydes, nitrates, phenols, nicotine

## Abstract

*Background.* Some electronic cigarette (EC) liquids of tobacco flavour contain extracts of cured tobacco leaves produced by a process of solvent extraction and steeping. These are commonly called Natural Extract of Tobacco (NET) liquids. The purpose of the study was to evaluate nicotine levels and the presence of tobacco-derived toxins in tobacco-flavoured conventional and NET liquids. *Methods*. Twenty-one samples (10 conventional and 11 NET liquids) were obtained from the US and Greek market. Nicotine levels were measured and compared with labelled values. The levels of tobacco-derived chemicals were compared with literature data on tobacco products. *Results.* Twelve samples had nicotine levels within 10% of the labelled value. Inconsistency ranged from −21% to 22.1%, with no difference observed between conventional and NET liquids. Tobacco-specific nitrosamines (TSNAs) were present in all samples at ng/mL levels. Nitrates were present almost exclusively in NET liquids. Acetaldehyde was present predominantly in conventional liquids while formaldehyde was detected in almost all EC liquids at trace levels. Phenols were present in trace amounts, mostly in NET liquids. Total TSNAs and nitrate, which are derived from the tobacco plant, were present at levels 200–300 times lower in 1 mL of NET liquids compared to 1 gram of tobacco products. *Conclusions*. NET liquids contained higher levels of phenols and nitrates, but lower levels of acetaldehyde compared to conventional EC liquids. The lower levels of tobacco-derived toxins found in NET liquids compared to tobacco products indicate that the extraction process used to make these products did not transfer a significant amount of toxins to the NET. Overall, all EC liquids contained far lower (by 2–3 orders of magnitude) levels of the tobacco-derived toxins compared to tobacco products.

## 1. Introduction

Electronic cigarettes (ECs) are becoming increasingly popular, with millions of users both in the US and in Europe [1,2,3]. These battery-powered devices deliver nicotine, although at a slower rate compared to tobacco cigarettes [4], and deal with the psycho-behavioural aspect of the addiction to smoking [5,6]. Due to these unique features they have the potential to serve as a valuable tobacco harm reduction product [7], by substituting tobacco cigarette consumption.

EC liquids consist mainly of propylene glycol, glycerol, nicotine and flavourings. Different flavour types are available, such as tobacco, sweets, fruits, beverages and nuts. Studies have shown that users frequently switch between flavours, while choices differ according to the duration of smoking substitution with EC use with tobacco flavours being more popular at EC use initiation [8]. In many cases, Generally Recognized As Safe (GRAS) flavour compounds for food are used [9], even for tobacco flavoured liquids. In other cases, industrially-produced tobacco absolute (used in the fragrance industry) is used, in an attempt to better simulate the tobacco flavour [10]. Additionally, there are cases of companies which produce their own (in-house) tobacco flavours by obtaining cured tobacco leaves from which an extract is produced, usually through solvent extraction and a steeping process [9]. These are commonly called Natural Extracts of Tobacco (NET). The main reason for their existence is anecdotal reports from EC consumer forums that they more accurately simulate the flavour of tobacco cigarettes and are used by consumers who prefer such flavour. A cytotoxicity study evaluated four NET samples and found that the aerosol of these liquids had cytotoxic properties on cultured cells, although at levels significantly lower compared to tobacco cigarette smoke [9]. It is unknown whether the use of NET leads to the delivery of toxic chemicals to the EC liquid, derived from the tobacco plant during the extraction process. Therefore, the purpose of this study was to evaluate the presence of selected chemicals derived from tobacco in NET EC liquids, and compare their levels with those present in liquids prepared with conventional (food GRAS or industrial tobacco absolute) flavourings. The focus of the study was to evaluate accuracy in nicotine labelling and content of tobacco-specific nitrosamines (TSNAs) and nitrates (which are present in the tobacco plant), phenols (which may be derived from heating cured tobacco leaves during flavour extraction) and aldehydes (which may be both present in the tobacco plant and derived from heating). Finally, since ECs are potential tobacco harm reduction products, a relevant comparison with tobacco products was considered appropriate. Therefore, we compared the levels of TSNAs and nitrate in EC liquids with literature data on tobacco products, and the levels of phenols with literature data on mainstream tobacco cigarette smoke.

## 2. Materials and Methods

### 2.1. Sample Selection

For the purpose of the study, EC liquids with tobacco flavour that were prepared using conventional flavour ingredients and NET-flavoured liquids were obtained from EC physical and internet shops. Information about the use of NET was obtained from the websites (internet shops) of the vendors. Unfortunately, no manufacturer (to the best of our knowledge) publicly reports the use of industrially produced tobacco absolute in their liquids, so, any liquid prepared without the use of NET was considered a conventional sample. Samples of conventional EC liquids were selected from the Greek market (manufactured in Greece and in Italy, all 10 samples). Samples using NET flavourings were obtained from the Greek (two samples, manufactured in the UK) and the US market (nine samples). In total, 21 samples were collected: 10 samples using conventional flavouring ingredients and 11 samples using NET. The samples were bought anonymously through e-shops or physical stores, and were immediately sent to the laboratory for analysis. All samples were refill (ready-to-use) liquids, and one bottle per liquid was tested.

### 2.2. Chemical Analysis

All methods used for this study were validated for linearity, recovery, precision and limits of detection in the EC sample matrix before use.

#### 2.2.1. Nicotine

Nicotine calibration standards were prepared over a range of 100–2000 μg/mL in 2-propanol, with n-heptadecane as an internal standard. All EC samples were analysed at a 50-fold dilution in 2-propanol with n-heptadecane added. A Hewlett Packard Model 5890 Series II GC (Hewlett Packard, Santa Clara, CA, USA) was equipped with an FID and a Restek Stabilwax column 30 m × 0.32 mm × 1.0 µm. The temperature program was: 60 °C for 1 min, 20 °C/min to 240 °C for 2 min.

The materials used for the GC analysis were: 2-propanol (low water): Fisher Scientific (Waltham, MA, USA); n-heptadecane (99% CAS 629-78-7): Sigma-Aldrich (St. Louis, MO, USA); nicotine (≥99% CAS 54-11-05): Sigma-Aldrich.

#### 2.2.2. TSNAs

Calibration standards for N-nitrosonornicotine (NNN) and 4-(methylnitrosamino)-1-(3-pyridyl)-1-butanone (NNK) were prepared over a range of 1–500 ng/mL in water, with NNN-d4 and NNK-d4 included as internal standards. The EC liquid samples were analysed at an 11-fold dilution in water and NNN-d4 and NNK-d4 added. Aliquots of the samples and standards were solvent-exchanged using SLE + cartridges (Biotage, Uppsala, Sweden) and eluted with methylene chloride. An Agilent 7890 GC coupled to an Agilent 7000 GC-MS Triple Quad mass spectrometer (Agilent, Santa Clara, CA, USA) was used for analysis. Separation was performed on an Agilent HP-5MS UI 30 m × 0.25 mm × 0.5 µm column, using helium as the carrier gas at 1.2 mL/min. A 5 µL injection was performed with the multimode inlet in PTV Solvent Vent mode. Initial inlet temperature was −10 °C, held for 2 minutes, then increased at 600 °C/min to 325 °C and held for the remainder of the run. The oven was operated at 35 °C for 2 min, then 50 °C/min to 230 °C for 5 min. The mass spectrometer was operated in positive chemical ionization (PCI) mode using ammonia as the reagent gas. Parent/daughter transitions were *m/z* 178→148 and *m/z* 178→120 for NNN, *m/z* 182.1→152.1 and *m/z* 182.1→124 for NNN-d4, *m/z* 208→122 and *m/z* 208→106 for NNK, and *m/z* 121→126 and *m/z* 212→152 for NNK-d4, with quantitation performed using the first transition listed for each compound. The limit of detection was 1ng/mL for both NNN and NNK. The materials used for the GC/MS/MS analysis were: deionized water, Millipore; methanol (Fisher OPTIMA**^®^**); methylene chloride (Fisher OPTIMA**^®^**); and ISOLUTE SLE + 1 mL supported liquid extraction cartridges (Biotage). Stock solutions of NNN (CAS 16543-55-8), NNN-d4 (CAS 66148-19-4), NNK (CAS 64091-91-4), and NNK-d4 (P/N 1707.10-K-AN) were purchased from Chiron (Trondheim, Norway).

#### 2.2.3. Nitrate

Standards were prepared over a range of 0.5–50 μg/mL in water. The EC liquid samples were analysed at a 50-fold dilution in water. An Agilent Model 1100, High Performance Liquid Chromatograph was equipped with a Dionex ED40 detector functioning in conductivity mode with a Thermo Fisher AS14 column. The mobile phase was 8mm sodium carbonate and 1 mm sodium bicarbonate with a flow rate of 1.2 mL/min. The limit of detection was 2.5 μg/mL.

The materials used for the HPLC analysis were: deionized water—Millipore (Billerica, MA, USA); Sodium Carbonate, 99.0%, Sigma-Aldrich (P/N S7795); Sodium bicarbonate, Sigma-Aldrich (P/N S014); Anion Mix, Accustandard (New Haven, CT, USA, P/N IC-MAN-10-R1-1).

#### 2.2.4. Phenols

The procedure followed was the HPLC phenol compound analysis method for mainstream cigarette smoke by the Cooperation Centre for Scientific Research Relative to Tobacco (CORESTA, Paris, France) [11], with the following modifications. Standards were prepared over a range of 0.05–10 μg/mL in mobile phase. All EC liquid samples were analysed at a 10-fold dilution in mobile phase. An Agilent Model 1100, High Performance Liquid Chromatograph was equipped with a fluorescence detector operating at an excitation of 280 nm and an emission at 310 nm and a Phenomenex Luna PFP, 4.6 × 150 mm, 3u column. The limit of detection was 0.05 μg/mL for all phenols.

The materials used for the analysis were: deionized water, Millipore; methanol HPLC Grade, Sigma-Aldrich (P/N 34860); hydroquinone (CAS #123-31-9), Alfa Aesar (Ward Hill, MA, USA) P/N A11411); resorcinol (CAS #108-46-3), Sigma-Aldrich (P/N 398047); catechol (CAS #120-80-9), Alfa Aesar (P/N A10164); phenol (CAS #108-95-2), Alfa Aesar (P/N A15760); *m*-cresol (CAS #108-39-4), Sigma-Aldrich, (P/N C85727); *o*-cresol (CAS #95-48-7), Sigma-Aldrich (P/N C85700); *p*-cresol (CAS #106-44-5), Alfa Aesar (P/N A13531).

#### 2.2.5. Formaldehyde and Acetaldehyde

The procedure followed was the HPLC carbonyl compound analysis method for mainstream cigarette smoke, by CORESTA [12], with the following modifications. Standards were prepared over a range of 0.1–20 μg/mL All EC samples were analysed at 11.5-fold dilution. An aliquot of the sample was combined with the 2,4-dinitrophenylhydrazine (DNPH) trapping solution and allowed to derivatize for 20 min, then quenched with 0.050 mL of pyridine. An Agilent Model 1100, High Performance Liquid Chromatograph was equipped with an Ultraviolet (UV) Detector operating at 365 nm and a Supelco Ascentis Express C18, 3.0 × 75 mm column. The limit of detection was 0.05 μg/mL for all carbonyl compounds. The materials used for the HPLC analysis were: deionized water, Millipore; phosphoric acid (H3PO4), 85%, A.C.S Reagent, Sigma-Aldrich (P/N 438081) (CAS #7664-38-2); DNPH (50%), TCI America (Portland, OR, USA) P/N D0845); acetonitrile (CAS #75-05-8), HPLC grade, Fisher (P/N LS121); tetrahydrofuran (CAS #109-99-9), HPLC grade, Fisher (P/N T427); isopropanol (CAS #67-63-0), distilled-in-glass, Fisher (P/N A464); pyridine, (CAS #110-86-1), Sigma-Aldrich (P/N 270407); acetaldehyde-2,4-DNPH, (CAS #1019-57-4), Sigma Aldrich (P/N 442434); formaldehyde-2,4-DNPH, (CAS #1081-15-8), Sigma-Aldrich (P/N 442597).

### 2.3. Statistical Analysis

For chemicals that were below the limit of detection (LOD), a value of LOD/2 was used for statistical comparisons. Data distributions were examined by a Kolmogorov-Smirnov test, after substituting <LOD with LOD/2. Only nicotine data were normally distributed. Continuous variables were expressed as mean (SEM) or median (IQR). Differences in the measurements between the 2 groups were evaluated by independent-samples *t*-test or Mann-Whitney U test. Comparison between the labelled and the measured level of nicotine was performed by paired samples *t*-test, while the % deviation from labelled nicotine concentration was compared between conventional and NET liquids by using independent samples *t*-test. No statistical comparison between conventional and NET liquids was performed for chemicals which were detected >LOD in less than half of the samples in one of the groups. Comparison between EC liquids and literature data on tobacco products were performed by Mann-Whitney U tests; the median (IQR) was computed from the reported levels per sample in he studies used for the comparison. Additionally, all samples in our study with levels <LOD were considered as having levels of LOD/2. A two-tailed *p* value of <0.05 was considered significant, and analysis was performed by commercially available software (SPSS v. 18, Chicago, IL, USA).

## 3. Results

### 3.1. Liquid Sample Analysis

The results of the chemical analysis are displayed in Table 1 and Table 2. On average, nicotine concentrations were similar to those labelled (paired *t*-test *p* = 0.092). Twelve samples were within 10% of the labelled value. Nine samples contained lower and 12 samples contained higher nicotine levels than labelled. Deviation from the labelled value ranged from −21% to 22.1%, with three samples exceeding 20% absolute deviation. No difference was found between groups in the deviation from labelled nicotine concentration.

Trace levels of TSNAs were found in all samples. In six samples, NNN was <LOD (three conventional and three NET samples), while NNK was detected in all samples. Higher levels of NNN and total TSNAs were observed in NET liquids, but the differences were not statistically significant (*p* = 0.141 for NNN, *p* = 0.549 for NNK and *p* = 0.197 for total TSNAs).

Nitrate was predominantly found in NET samples, with only two of them being nitrate-free. On the contrary, only two conventional samples contained detectable levels of nitrates.

Small amounts of phenols were detected in nine samples, seven of which were NET liquids. Catechol was detected in two NET samples. Two conventional and four NET samples contained *m*-cresol and *o*-cresol, with higher levels observed in NET liquids. *p*-Cresol was present in one conventional and three NET samples. Phenol was present in one conventional and four NET samples. Hydroquinone and resorcinol were not detected in any sample. Total phenols were higher in NET liquids (1.5 [0.2–4.1] μg/mL *vs.* 0.2 [0.2–1.7] μg/mL), but the difference was not statistically significant (*p* = 0.101).

Acetaldehyde was detected in all but 3 conventional samples but only in three NET samples. Formaldehyde was present in all but one sample. The levels of formaldehyde were similar in the two groups (*p* = 0.314).

**Table 1:** Nicotine and tobacco-specific nitrosamines in electronic cigarette liquids produced with conventional flavours or natural extracts of tobacco (NET). Deviation from labelled nicotine level is also displayed.

	Labelled Nicotine (mg/mL)	Measured Nicotine (mg/mL)	Nicotine Deviation (%)	NNN (ng/mL)	NNK (ng/mL)	Total Nitrosamines (ng/mL)
**Limits of detection**		0.5		1.0	1.0	1.0
**Conventional liquids**						
AtmosLab Bal	18	21.6	20.1	<LOD	5.2	5.2
AtmosLab RY69	18	22	22.1	5.1	9.9	15.0
ElGreco Americano	18	17.6	−2.0	<LOD	1.7	1.7
ElGreco City	18	17.3	−3.9	<LOD	5.5	5.5
ElGreco Classic	18	18.2	0.9	<LOD	2.5	2.5
Flavourart MaxBlend	18	16.9	−6.2	2.0	5.8	7.7
Flavourart RY4	18	17.8	−1.0	17.3	22.4	39.7
Flavourart Virginia	18	19.9	10.7	4.1	4.1	8.2
Nobacco American Tobacco	18	21	16.4	1.6	3.4	5.0
Nobacco Golden Margy	12	12.2	1.6	<LOD	3.6	3.6
**Average ^a^**	17.4 (0.6)	18.5 (0.9)	5.9 (3.3)	1.3 (0.5–4.4)	4.7 (3.2–6.8)	6.1 (3.8–9.9)
**NET liquids**						
Cravin Vapes BOMB	12	10.5	−12.8	6.4	3.7	10.1
Cravin Vapes Perique	12	10.8	−9.8	12.5	5.4	18.0
ElToro Cigarrillos	18	19.8	10.1	<LOD	2.6	2.6
ElToro Puros	24	25.8	7.6	<LOD	2.5	2.5
MOV FullVirginiaFlake	18	19	5.4	<LOD	3.2	3.2
MOV Pendragon	18	19.2	6.4	11.3	10.8	22.1
MOV Southern Gentleman	18	17.3	−3.9	16.7	9.2	25.9
Naturally Extracted Tobacco Big Spirit	12	11.6	−3.6	1.6	4.6	6.3
Naturally Extracted Tobacco NS Dark	12	9.5	−21.0	9.5	6.3	15.8
QuickNicJuice Grandpa’s Night Cap	12	14.3	18.8	22.9	15.5	38.5
QuickNicJuice Hump Back	12	14.3	19.2	16.8	15.1	31.9
**Average ^a,b^**	15.3 (1.2)	15.6 (1.5)	1.5 (12.9)	9.5 (0.5–16.7)	5.4 (3.2–10.8)	15.8 (3.7–25.9)

NNN, N-nitrosonornicotine; NNK, 4-(methylnitrosamino)-1-(3-pyridyl)-1-butanone; LOD, limit of detection; NET, natural extract of tobacco. **^a^** Average presented as mean (SEM) or median (interquartile range). To obtain average values, samples with levels <LOD were considered as containing LOD/2. **^b^** No statistically significant differences between groups were observed.

**Table 2:** Level of nitrates, phenols and aldehydes in electronic cigarette liquids produced with conventional flavours or natural extracts of tobacco (NET). Hydroquinone and resorcinol were not detected in any of the samples.

	Nitrate (μg/mL)	Catechol (μg/mL)	*m*-Cresol (μg/mL)	*o*-Cresol (μg/mL)	*p*-Cresol (μg/mL)	Phenol (μg/mL)	Acetaldehyde (μg/mL)	Formaldehyde (μg/mL)
**Limits of detection**	2.5	0.05	0.05	0.05	0.05	0.05	0.12	0.12
**Conventional liquids**								
AtmosLab Bal	<LOD	<LOD	<LOD	<LOD	<LOD	<LOD	1.82	2.53
AtmosLab RY69	<LOD	<LOD	<LOD	<LOD	<LOD	<LOD	20.06	2.14
ElGreco Americano	7.5	<LOD	<LOD	<LOD	<LOD	<LOD	<LOD	2.91
ElGreco City	<LOD	<LOD	<LOD	<LOD	<LOD	<LOD	2.55	<LOD
ElGreco Classic	<LOD	<LOD	<LOD	<LOD	<LOD	<LOD	0.75	3.95
Flavourart MaxBle< LOD	<LOD	<LOD	0.32	4.40	<LOD	1.42	5.23	6.21
Flavourart RY4	<LOD	<LOD	<LOD	<LOD	<LOD	<LOD	3.61	0.73
Flavourart Virginia	<LOD	<LOD	<LOD	<LOD	<LOD	<LOD	<LOD	3.24
Nobacco American Tobacco	<LOD	<LOD	<LOD	<LOD	<LOD	<LOD	2.42	1.99
Nobacco Golden Margy	15.4	<LOD	0.45	5.46	0.69	< LOD	<LOD	1.94
**Average ^a^**	-	-	-	-	-	-	2.1 (0.1–4.0)	2.3 (1.6–3.4)
**NET liquids**								
Cravin Vapes BOMB	47.6	<LOD	<LOD	<LOD	<LOD	<LOD	<LOD	1.75
Cravin Vapes Perique	15.2	<LOD	<LOD	<LOD	<LOD	0.80	<LOD	2.31
ElToro Cigarrillos	<LOD	<LOD	<LOD	<LOD	<LOD	1.30	1.46	1.71
ElToro Puros	<LOD	<LOD	0.13	0.16	0.22	2.29	1.73	2.10
MOV FullVirginiaFlake	15.2	<LOD	5.31	1.40	<LOD	<LOD	<LOD	27.95
MOV Pe< LODragon	145.2	<LOD	3.75	0.22	<LOD	<LOD	<LOD	4.77
MOV Southern Gentleman	163.8	1.71	0.83	0.35	0.87	3.65	<LOD	2.29
Naturally Extracted Tobacco Big Spirit	11.5	<LOD	<LOD	<LOD	<LOD	<LOD	0.45	4.96
Naturally Extracted Tobacco NS Dark	159.9	<LOD	<LOD	<LOD	<LOD	<LOD	<LOD	3.45
QuickNicJuice Gra< LODpa’s Night Cap	317.9	1.71	<LOD	<LOD	1.03	<LOD	<LOD	4.28
QuickNicJuice Hump Back	32.6	<LOD	<LOD	<LOD	<LOD	<LOD	<LOD	3.17
**Average ^a^**	32.6 (11.5–159.9)	-	-	-	-	-	-	3.2 (2.1–4.8) **^b^**

LOD, limit of detection; NET, natural extract of tobacco. **^a^** Average presented as mean (SEM) or median (interquartile range). To obtain average values, samples with levels <LOD were considered as containing LOD/2. No average was calculated for chemicals which were detected in less than half of the samples. **^b^** No statistically significant difference between groups was observed.

### 3.2. Comparison with Tobacco Products

The results of this study concerning chemicals present in tobacco plant (TSNAs and nitrate) were compared with literature data on tobacco products. A study by Stepanov *et al.* measured NNN and NNK by Gas Chromatography in 16 samples of four brands of tobacco cigarettes and reported the results in amount per gram of tobacco [13], while CORESTA reported the results of nitrate levels per gram of tobacco in six cured tobacco samples [14]. The results of the comparison between levels per mL EC liquid and levels per gram of tobacco are displayed in Table 3. The average levels of NNN, NNK, total TSNAs and nitrate in all EC liquids were >1400, >100, >400 and >1300 times lower compared to tobacco respectively (*p* < 0.001 for all). For NET liquids alone, the respective levels were >250, >140, >200 and >300 times lower (Table 3). Phenols are present mostly in tobacco cigarette smoke, derived from heating of polyphenols present in the tobacco plant [15]. Therefore, a comparison between 1 mL of EC liquids and the smoke of one tobacco cigarette was performed, using the findings from analysis of seven commercial cigarette samples smoked under Health Canada Intense puffing regime by CORESTA using HPLC [11]. Total phenols were present at levels 1200 times lower in all EC liquids, and 160 times lower in NET liquids compared to tobacco cigarette smoke (Table 3).

**Table 3:** Difference between tobacco cigarette products and electronic cigarette liquids selected tobacco-derived toxins. Statistically significant differences were found for all analyses (*p* < 0.001).

	Tobacco Products (per g Weight)	EC Liquids (per mL) ^a^	Ratio ^b^	NET Liquids (per mL)	Ratio ^c^
NNN (ng)	2750 (2125–2975)	1.9 (0.5–11.9)	1447	9.5 (0.5–16.7)	289
NNK (ng)	760 (552–1140)	5.2 (3.3–9.5)	146	5.4 (3.2–10.8)	141
Total nitrosamines (ng)	3440 (2833–3808)	7.7 (3.9–20.0)	447	15.8 (3.7–25.9)	218
Nitrate (μg)	10200 (1975–14700)	7.5 (1.3–40.1)	1360	32.6 (11.5–159.9)	313
Total phenols (μg) **^d^**	240 (127–252)	0.2 (0.2–3.5)	1200	1.5 (0.2–4.1)	160

EC, electronic cigarettes; NNN, N-nitrosonornicotine; NNK, 4-(methylnitrosamino)-1-(3-pyridyl)-1-butanone. Data presented as median (interquartile range) **^a^** Average of all EC liquids tested (both conventional and NET liquids). **^b^** Ratio of tobacco products divided by average from all EC liquids. **^c^** Ratio of tobacco products divided by average from NET liquids. **^d^** Phenols detected in the smoke of one tobacco cigarette.

## 4. Discussion

This is the first study to evaluate a specific group of EC liquids, using cured tobacco leaves to extract the flavouring (NET liquids), for the presence of selected tobacco-derived toxins. None of the liquid samples was free from potentially harmful chemicals. Compared to conventional liquids, levels of TSNAs and formaldehyde were similar in NET liquids, as was the deviation from labelled nicotine content of the samples. Phenols were more prevalent in NET liquids, while acetaldehyde was found predominantly in conventional liquids. A characteristic finding in NET liquids was the nearly universal presence of nitrate. Of note, the levels of TSNAs and nitrate in EC liquids were 1 to 2 orders of magnitude lower compared to tobacco products.

Differences between nicotine content and labelling have been detected in previous studies. Kischner *et al.* found discrepancies from −50% to 42% in labelling compared to true content of refill liquids [16]. Similar results were reported in another recent study [17]. Davis *et al.* found that 46 out of 50 liquids contained higher than labelled nicotine concentration [18]. Our results are in agreement with a study by Etter *et al.* who found that the deviation from the label ranged from ‒15% to 21% [19]. Moreover, almost half (43%) of the samples tested herein contained lower than labelled nicotine concentrations. Interestingly, we did not detect any difference between NET and conventional liquids in deviation of nicotine levels from the label, indicating either that the flavour extraction methods used do not extract nicotine from the tobacco leaves or that manufacturers of NET liquids may compensate for any nicotine being present in the flavouring extract in the formulation process.

TSNAs are probably the most important compounds associated with negative health effects in tobacco cigarettes, mostly due to a combination of abundance and strong carcinogenicity [20,21]. They are present in very high quantities in both tobacco cigarette and smokeless tobacco products (in μg/g of tobacco weight) [13]. Herein, the levels found were traces, in ng/mL range, verifying previous observations [7,22,23]. No statistically significant difference was observed between NET and conventional liquids in TSNAs levels; three of the five samples with the lowest levels of nitrosamines were in fact NET liquids. Although not studied until now, it is unlikely that nitrosamines are additionally produced and emitted in EC aerosol during the evaporation process. Goniewicz *et al.* evaluated nitrosamine levels in the aerosol of 12 EC products, and found levels similar to our study [24].

Nitrate and aldehydes are compounds with significant toxic and carcinogenic potential. Nitrate is converted to nitrite in saliva [25] which can participate in the endogenous production of TSNAs [26]. A characteristic finding of this study was that nitrate was almost exclusively found in NET liquids, therefore, we can conclude that they are derived from the flavour extraction process. Still the levels were much lower compared to tobacco products. Acetaldehyde and formaldehyde were present in a substantial proportion of liquids, both conventional (both compounds) and NET samples (predominantly formaldehyde). These chemicals are also present in tobacco products but at much higher levels compared to EC liquids. It should be mentioned that acetaldehyde is a GRAS substance for use in food (FEMA Nr 2003), therefore, it is possible that the source of acetaldehyde is food flavourings used in conventional EC liquids. However, acetaldehyde is classified as a possible human carcinogen (Group 2B) by the International Agency of Research on Cancer [27], and every effort should be made to avoid the presence of acetaldehyde in EC liquids.

Phenols are compounds with significant genotoxic, cardiotoxic and carcinogenic properties. They are mostly present in tobacco cigarette smoke rather than tobacco leaves [28]. Phenols were detected in nine of the 21 samples tested (four conventional and five NET liquids), but none of them contained all the phenols tested. It is known that phenols may be produced from heating tobacco; therefore, it is possible that in some cases tobacco leaves are heated during the extraction process. Still, the levels present in the liquids tested were much lower compared to the levels found in tobacco smoke. It remains to be seen if phenols may be additionally produced from ECs during the evaporation process.

The results of the study indicate that a proportion of conventional liquids were also contaminated with tobacco-derived chemicals. Besides TSNAs, which may be derived at low levels from pharmaceutical grade nicotine and are also present in nicotine replacement therapy products [7], nitrate and phenols were found in a limited number of samples. Although compounds approved for food use are commonly used as flavourings in conventional liquids, several of them also use industrially-produced tobacco absolute (commonly used in fragrances) to imitate the tobacco flavour. Therefore, that could potentially be the source for the phenols and nitrate found in these liquids. To the best of our knowledge, companies do not usually mention if tobacco absolute is used in their flavours. We propose that this should be mentioned in the labelling, since tobacco absolute is not approved for food use and it may be the source of exposure to some additional toxic chemicals compared to liquids not using it.

Two of the NET samples evaluated in this study were previously examined in aerosol form to determine their cytotoxic properties on cultured cardiomyoblasts [8]. They were found to be cytotoxic, although at levels significantly lower compared to tobacco cigarette smoke. Interestingly, these samples showed a lower chemical constituent profile in the testing herein; in particular, they contained very low levels of TSNAs and no nitrate, while levels of aldehydes were similar to conventional liquids. They both contained phenols, although at very low levels. It is probable that some other chemicals, not evaluated in this study, may be responsible for the cytotoxic properties.

Certain limitations apply to this study. Firstly, only one sample per liquid was tested, therefore, we could not assess the inter-batch variability. Depending on the quality and consistency of the production process, it is possible that significant differences between batches may exist. This should be further explored. Moreover, a larger selection of samples would increase the statistical power of the comparisons, especially in the cases of NNN, total TSNAs and total phenols which were found at higher levels in NET compared to conventional liquids but the differences were not statistically significant. Still, the levels were very low in both groups compared to tobacco products. Formaldehyde and acetaldehyde are formed during the heating process of EC aerosol production [24]. Thus, the levels reported herein underestimate true exposure to the consumer. However, we have determined that another source of aldehydes is the liquid itself. This should be considered when assessing aldehyde emissions to the aerosol, and it is necessary to evaluate the presence of these compounds in the liquid used to produce the aerosol. Recent studies have detected aldehyde levels in the aerosol approximating [29] or exceeding [30] the levels found in tobacco smoke. Such levels are probably not affected by the presence of trace amounts of aldehydes in the liquid as found herein. However, a major pitfall in laboratory evaluation of aerosol chemistry is that overheating of the liquid, resulting in the dry puff phenomenon [31], cannot be detected; thus, the findings may not be associated with relevant exposure of user during normal daily use, and this should be addressed in future studies. Finally, the analysis focused on EC liquids and not on aerosol. Although unlikely, it is currently unknown whether TSNAs and nitrate are produced during the heating and evaporation of the EC liquid; this should be explored through studies of aerosol chemistry.

## 5. Conclusions

In this study, EC liquids were evaluated for the presence of selected tobacco-derived chemicals. A specific category of liquids, produced by extracting flavour from cured tobacco leaves, was evaluated and compared with conventional liquids of tobacco flavour. Nicotine content did not deviate by more than 22% in any liquid, with more than half of them being within the 10% range which is accepted for pharmaceutical products. None of the liquids was free from potentially harmful chemicals. NET liquids could result in exposure to somewhat higher levels of toxins compared to conventional EC liquids, especially for nitrate and phenols. Major tobacco-derived toxins, such as TSNAs and nitrates, were present at very low levels compared to tobacco products. Further studies should evaluate whether these chemicals are emitted to the aerosol, while clinical studies will determine whether the levels of toxins found in EC liquids and aerosol are associated with adverse health effects.
